# Multiple Urethral Stricture and Urinary Fistulæ—Divulsion and Urethrotomy

**Published:** 1877-09

**Authors:** Alexander Fisher


### MULTIPLE URETHRAL STRICTURE AND URINARY FISTULÆ—DIVULSION AND URETHROTOMY.

By Alexander Fisher, M. D.

On the 12th of February, 1877, I was called to attend Mr. J., aged 29 years, of a bilious temperament, and he gave me the following history of his case:

Up to the year 1862, he enjoyed fair health, but at the date mentioned, he contracted preputial chancre, which was not treated for nearly one year, the sore discharging most of the time. He then consulted a physician, under whose treatment the sore healed, but in December of 1863 he contracted a gonorrhœa, which lasted for a long time, resulting in a gleet which had never been relieved. In 1871, he first discovered that he could not pass his urine freely, and his physician decided that he had a stricture, though the urethra was not explored, and internal treatment only employed. The straining and dysuria, with the occasional escape of blood and mucus from the urethra, continued without much impairment of the general health, until January, 1875, when the perineum became inflamed, and swelled till a tumor presented as large as the fist, which was opened and gave exit to about a pint of pus. Two days afterward, more than half the quantity of urine excreted passed through the opening, though when making expulsive efforts some water still passed by the urethra. In December, 1875, he had a discharging sore over the tibia of the right leg, which remained open for about one year, and finally closed under the administration of the iodide of potassium, leaving a delicate, slightly pigmented, circular and unattached cicatrix, two inches in diameter.

In June, 1875, the perineal abscess healed, and the urine passed normally for a few months, but in January, 1876, the perineum and posterior scrotal region became again swollen, and opened in several places, through which urine escaped, in quantity about two-thirds of that evacuated.

At the time of my first examination, he told me that he had a chill every few days, and that he had been thus affected for about two years. His countenance was sallow, skin cool and clammy, tongue coated, bowels constipated, no appetite. In the perineal region, I found a tumor as large as the fœtal head at term, having a transverse diameter of about five inches, and a vertical diameter of six inches, involving the scrotum and perineum, and projecting forwards between the thighs, so as to interfere with the gait. The perineal portion of this was of a stony hardness; the epidermis covering it was hypertrophied, and, at irregular distances upon its surface, could be seen ten or twelve nipple-shaped protuberances containing patulous or closed orifices of as many urethral fistulæ, some in the process of healing and others discharging pus and urine. These gave exit together to more than two-thirds of all the urine secreted; that which escaped from the meatus, only followed severe straining, and was evacuated in a thread-like stream.

Upon exploration of the urethra, I discovered three distinct strictures—one at the meatus, another, commencing about two and a half inches from the meatus, extending to a depth of two inches in the pendulous urethra; a third in the bulbo-membranous portion of the canal, about one inch in length. All these were extremely dense and resilient, and the deepest was so contracted, that after a filiform bougie was introduced, it was grasped tightly in the canal. The stricture at the meatus was so narrow that it would barely admit with force a No. 9 F. bougie. This I incised at once with a bistoury.

He had been taking large doses of the iodide of potassium, and these had produced an intense repugnance to the drug, which had been administered with a view to producing resorption of the tumor. I ordered him to take the muriated tincture of iron with strychnia, and quinia in quantity sufficient to control the chills. His health speedily improved, though he could walk only with difficulty in consequence of the separation of the thighs, necessitated by the tumor.

I endeavored to enter the bladder with a great variety of instruments; these were all arrested, however, at the deepest of the strictures. After perseverence for many hours (at different sittings) a slender, filiform bougie of whalebone was finally seen to escape from one of the fistulous openings in the tumor; evidently the urethral orifice of this fistulous tract had produced a want of correspondence between the portions of the deep urethra on either side of it. After many fruitless attempts to enter the bladder with this instrument, I told the patient, who was a remarkably intelligent artist, to make the attempt himself in the interval of my visits. He had the good fortune to slip the instrument into the bladder on the 24th of March, and at once summoned me. The patient was fully anaesthetized with chloroform by Dr. Hyde, when we threaded the whalebone bougie (which had been carefully held in place ever since its passage into the bladder) into the eye of Thompson's divulsor, and passed the instrument into the bladder, making use of great force, and yet feeling sure that the divulsor followed the guide. Upon widest dilitation of this instrument the strictures did not split, and as a result the operation was bloodless. We then introduced Holt's divulsor, and no distension that this instrument was capable of making, was sufficient to overcome the resiliency of the contracted portion. As a result of the operation the patient became more comfortable, and much less urine flowed through the fistulous openings, but in three days afterward, we could not pass the smallest instrument into the bladder.

In consequence of the general improvement of the patient's condition, and as there was no special urgency in the case, I deferred operating a second time, until the recovery from the effect of the first was complete. On the 31st of March, after making ineffectual attempts for half an hour to introduce a second time the filiform bougie, it again entered the bladder, and the patient being completely anæsthetized by Dr. Hyde, I again threaded Thompson's divulsor upon the bougie, passed it into the bladder, and fully dilated the canal. Immediately thereafter I introduced Maisonneuve's urethrotome, and completely divided the urethra longitudinally from the meatus to the anterior border of the prostate gland, when a No. 14 (A) sound was readily passed into the bladder.

The next morning the urine was freely and entirely passed through the urethra, and the patient stated that he had not made water so freely in ten years. From that time the fistulous openings in the perineal tumor ceased giving exit to a purulent discharge, and soon commenced to close; while the tumor itself subsided gradually till at this date, May 21st, it is not so large as a goose's egg and is daily growing smaller. The patient has gained twenty-five pounds in actual weight, and expresses himself as feeling quite restored in health-

His only treatment has been the administration of iron and strychnia, with quinine to counteract the miasmatic element in his case, and the repeated introduction of the steel sound in order to insure the continued permeability of the urethra.

The interesting features in this case, which are worthy of note, are, 1st. The possibility of entering with filiform bougies through a canal which is capable of giving exit to but a small moiety of the urine, and that only in a thread-like stream after expulsive efforts; 2d. The small value of distending instruments in resilient strictures; and, 3d. The patience and perseverance which are required in order to pass even a filiform bougie through a stricture which seems to be impermeable.

